# Intestinal permeability – a new target for disease prevention and therapy

**DOI:** 10.1186/s12876-014-0189-7

**Published:** 2014-11-18

**Authors:** Stephan C Bischoff, Giovanni Barbara, Wim Buurman, Theo Ockhuizen, Jörg-Dieter Schulzke, Matteo Serino, Herbert Tilg, Alastair Watson, Jerry M Wells

**Affiliations:** Department of Nutritional Medicine/Prevention, University of Hohenheim, Fruwirthstrasse 12, Stuttgart, 70593 Germany; Department of Medical and Surgical Sciences and Centre for Applied Biomedical Research, University of Bologna, Bologna, Italy; Department of Surgery, Maastricht University Medical Centre, Maastricht, The Netherlands; Nutricom, Rumpt, The Netherlands; Department Gastroenterology, Division General Medicine & Nutrition, Charité Berlin, CBF, Germany; Institut National de la Santé et de la Recherche Médicale (INSERM) & Université Paul Sabatier (UPS), Unité Mixte de Recherche (UMR) 1048, Institut de Maladies Métaboliques et Cardiovasculaires (I2MC), Toulouse, France; Medical University Innsbruck, Department of Internal Medicine I, Innsbruck, Austria; Norwich Medical School, University of East Anglia, Norwich Research Park, Norwich, UK; Host-Microbe Interactomics Group, Animal Sciences Department, Wageningen University and Research Centre, Wageningen, The Netherlands

**Keywords:** Intestinal barrier, Intestinal permeability, Microbiota, Tight junctions, Obesity, Inflammatory bowel disease, Irritable bowel syndrome, Prebiotics, Probiotics, Gut health

## Abstract

Data are accumulating that emphasize the important role of the intestinal barrier and intestinal permeability for health and disease. However, these terms are poorly defined, their assessment is a matter of debate, and their clinical significance is not clearly established. In the present review, current knowledge on mucosal barrier and its role in disease prevention and therapy is summarized. First, the relevant terms ‘intestinal barrier’ and ‘intestinal permeability’ are defined. Secondly, the key element of the intestinal barrier affecting permeability are described. This barrier represents a huge mucosal surface, where billions of bacteria face the largest immune system of our body. On the one hand, an intact intestinal barrier protects the human organism against invasion of microorganisms and toxins, on the other hand, this barrier must be open to absorb essential fluids and nutrients. Such opposing goals are achieved by a complex anatomical and functional structure the intestinal barrier consists of, the functional status of which is described by ‘intestinal permeability’. Third, the regulation of intestinal permeability by diet and bacteria is depicted. In particular, potential barrier disruptors such as hypoperfusion of the gut, infections and toxins, but also selected over-dosed nutrients, drugs, and other lifestyle factors have to be considered. In the fourth part, the means to assess intestinal permeability are presented and critically discussed. The means vary enormously and probably assess different functional components of the barrier. The barrier assessments are further hindered by the natural variability of this functional entity depending on species and genes as well as on diet and other environmental factors. In the final part, we discuss selected diseases associated with increased intestinal permeability such as critically illness, inflammatory bowel diseases, celiac disease, food allergy, irritable bowel syndrome, and – more recently recognized – obesity and metabolic diseases. All these diseases are characterized by inflammation that might be triggered by the translocation of luminal components into the host. In summary, intestinal permeability, which is a feature of intestinal barrier function, is increasingly recognized as being of relevance for health and disease, and therefore, this topic warrants more attention.

**Electronic supplementary material:**

The online version of this article (doi:10.1186/s12876-014-0189-7) contains supplementary material, which is available to authorized users.

## Introduction

Why do we need a gut barrier? The intestinal barrier covers a surface of about 400 m^2^ and requires approximately 40% of the body’s energy expenditure. It prevents against loss of water and electrolytes and entry of antigens and microorganisms into the body [1] while allowing exchange of molecules between host and environment and absorption of nutrients in the diet. Specialized adaptations of the mammalian intestinal mucosa fulfill two seemingly opposing functions: firstly to allow a peaceful co-existence with intestinal symbionts without eliciting chronic inflammation and secondly to provide a measured inflammatory and defensive response according to the threat from pathogens [2],[3]. It is a complex multilayer system, consisting of an external "physical" barrier and an inner "functional" immunological barrier. The interaction of these 2 barriers enables equilibrated permeability to be maintained [4]. To understand this complex barrier, not only the functions of its components, but also the processes of interactions of bacterial and other luminal components with cells and receptors of the host needs to be considered. Experimental data showed that disruption of the peaceful co-existence with intestinal symbionts at early life, and possibly even later in life, results in severe immunodeficiency and risk of disease [5]-[7]. Such findings support the hypothesis that the breakdown of intestinal barrier control mechanisms means danger and possibly disease.

**Figure 1:**
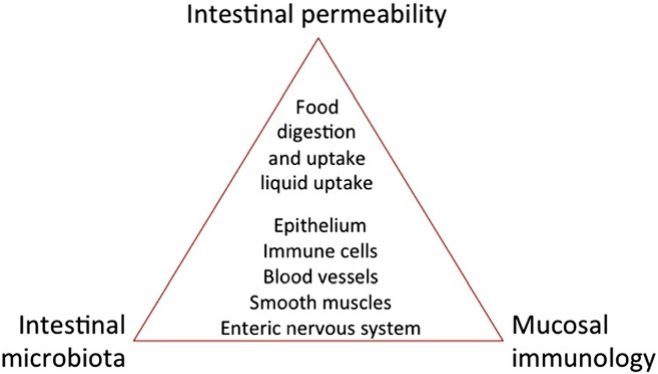
**Relation between intestinal permeability, intestinal microbiota, and mucosal immunology.** For details see text.

What is the difference between intestinal barrier and intestinal permeability? The two terms have been used synonymously although they probably do not mean the same thing. A clear definition of such parameters as means to assess them is mandatory to avoid future confusion and to assess their impact for disease prevention and disease. In fact, intestinal permeability is a barrier feature closely linked to the intestinal commensal microbiota as well as to the elements of the mucosal immune system (Figure 1). Many factors can alter intestinal permeability such as gut microbiota modifications, mucus layer alterations, and epithelial damage, resulting in translocation of luminal content to the inner layers of the intestinal wall. Moreover, lifestyle and dietetic factors like alcohol and energy-dense food can increase intestinal permeability such as alcohol and energy-dense Western style diet [8]-[10].

What is the clinical significance of the intestinal barrier and intestinal permeability? There is now increasing evidence for the notion that loss of intestinal barrier functions can occur either abruptly, e.g. following a major trauma resulting in gram-negative sepsis and multi-organ failure (MOF), or gradually leading to chronic inflammatory diseases. During the last 30 years, almost 2000 publications appeared according to the PubMed database with a linear increase from approximately 10 publications a year in the eighties of last century to almost 100 at present. Although we learned over the past decade about the link between the intestinal barrier and diseases, the mechanisms are not precisely understood. For example, we have limited knowledge of what causes initially intestinal barrier dysfunction, and what prevents or restores it. The former might involve different events including virus infections, reduced perfusion of the mucosa, drugs, or changes in the microbiota [6]. New data suggest that intestinal barrier and intestinal microbiota play a role in many different diseases such as idiopathic liver fibrosis or intestinal dysbiosis the mechanism of which were largely unclear until recently [11]-[13].

Understanding that the intestinal barrier also means to have clear definitions, clear modes of assessment *in vitro* and *in vivo* in animal models and in humans, and clear strategies of how to perform human trials in this field. These topics have been extensively discussed within an expert panel in Frankfurt/Germany in June 2012. The major results are summarized and extended in the following text.

## Review

### Definition of intestinal permeability

#### Definition of intestinal permeability and intestinal barrier

The term "mucosal barrier" was adopted by Cummings in 2004 to describe the complex structure that separates the internal milieu from the luminal environment [14]. The physical barrier includes a cellular component consisting of the vascular endothelium, the epithelial cell lining, and the mucus layer. Next to this physical barrier, chemical substances take part in the barrier function as well. They consist of digestive secretions, immune molecules, cell products like cytokines, inflammatory mediators and antimicrobial peptides, mainly produced by Paneth cells in the crypts of the small intestine. The intestinal microbiota is involved in metabolic processes and modulates the barrier, but does not represent a barrier function per se. On the other hand, the microbiota contributes to "intestinal health" - a term that is increasingly used although poorly defined. It may be described as a state of physical and mental well-being in the absence of gastro-intestinal complaints that require the consultation of a doctor, in the absence of indications of or risks for bowel disease and in the absence of confirmed bowel disease [6].

The terms “intestinal barrier” and “intestinal permeability” describe two different aspects of the same anatomical structure, the intestinal wall composed of four layers, the mucosa, the submucosa, the muscularis and the serosa. “Intestinal permeability” is a term shaped preferentially by electrophysiologists studying epithelial permeability in Ussing chambers using tissue explants from animals or humans for research purposes [15],[16]. By extrapolating the Ussing chamber experiments to the *in vivo* situations, particular permeability tests have been developed such as the sugar test [17]. All these tests have in common that defined molecules such as electrolytes or sugars of different molecular weight are used for their capacity to enter and cross the epithelium or the mucosal layer, respectively and finally entering the submucosal site (Ussing chamber) or the blood (sugar test).

“Intestinal barrier” is a term that has been established more recently by gastroenterologists, immunologists and microbiologists to emphasize the protective component of the gut shielding us against bacterial invasion, or invasion of other microorganisms and their toxins. Therefore, the means of assessing barrier functions were different to the approaches by the electrophysiologists and consisted of measuring translocation of bacteria or bacterial products like endotoxin from the gut into the portal vein, the liver or the systemic bloodstream. Likely, the mechanisms determining electrolyte fluxes, carbohydrate permeability and bacterial translocation are quite different; however, all approaches have in common that transfer of defined molecules across the intestinal wall (or parts of it) are measured. This knowledge might provide a basis for a definition of intestinal permeability, and also of normal and pathological intestinal permeability (Table 1).

**Table 1:** **Definitions**

***Intestinal barrier***	*is a functional entity separating the gut lumen from the inner host, and consisting of mechanical elements (mucus, epithelial layer), humoral elements (defensins, IgA), immununological elements (lymphocytes, innate immune cells), muscular and neurological elements*
***Intestinal permeability***	*is defined as a functional feature of the intestinal barrier at given sites, measurable by analyzing flux rates across the intestinal wall as a whole or across wall components of defined molecules that are largely inert during the process and that can be adequately measured in these settings*
***Normal intestinal permeability***	*is defined as a stable permeability found in healthy individuals with no signs of intoxication, inflammation or impaired intestinal functions*
***Impaired intestinal permeability***	*is defined as a disturbed permeability being non-transiently changed compared to the normal permeability leading to a loss of intestinal homeostasis, functional impairments and disease*

Thus, according to the proposed definitions, intestinal permeability can be understood as a measurable feature of the intestinal barrier. The proposed definitions are related to above mentioned definition of gut health thought to be closely related to the intestinal barrier and intestinal permeability [1],[6].

The proposed definitions extent the more descriptive approach by Cummings et al. [14], who summarized: (1) The mucosal barrier is a complex structure that separates the internal milieu from the luminal environment. (2) Physically, the barrier includes cellular and stromal components, from the vascular endothelium to the epithelial cell lining, and the mucus layer, which consists of a gel formed by the interaction of various mucosal secretions, namely mucins, trefoil peptides and surfactant lipids. (3) Apart from the physical barrier, a chemical barrier exists consisting of digestive secretions, antimicrobial peptides, and other cell products (cytokines, inflammatory mediators etc.). (4) Also the intestinal microbiota can be considered as a barrier. (5) Finally immune functions and motility contribute to the barrier.

### Key element of the intestinal barrier affecting intestinal permeability

**Figure 2:**
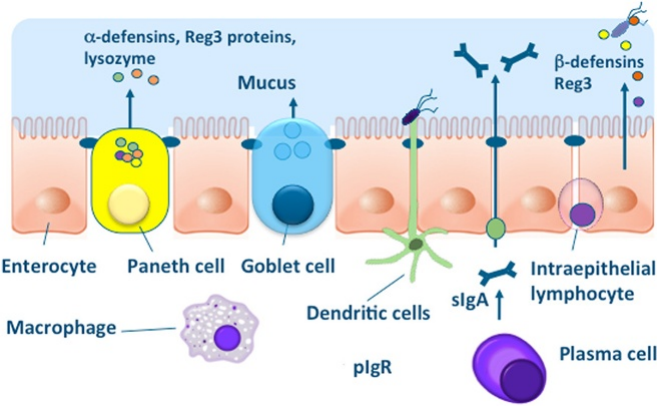
**Chemical and physical barriers in the intestine.** For explanations see text.

In recent times, several important molecules and mechanisms of the intestinal barrier could be identified (Figure 2). A single layer of epithelial cells form the main physical barrier between the lumen and mucosal tissues. The paracellular space is sealed by tight junctions (TJ) which regulate the flow of water ions and small molecules through the composition of claudins and other proteins in the junctional complex [5],[18],[19]. Below the tight junctions are the adherence junctions (AJ), which are important in cell-cell signaling and epithelial restitution as well as desmosomes supporting epithelial stability. TJ complexes consist of intra-membrane proteins, occludin and different members of the claudin family depending on the tissue and location that interlink within the paracellular space. Occludin and claudins and tricellulin link adjacent cells to the actin cytoskeleton through cytoplasmatic scaffolding proteins like Zonula occludens proteins [5]. Tricullin and occludin as well as a new protein named marvelD3, can be replaced in part by each other, but if all three are down-regulated or lacking, severe leakage occurs [20]. Claudins are a family of tight junction proteins consisting of sealing molecules and pores facilitating water and electrolyte loss. Zonula occludens proteins (ZO-1, ZO-2 and ZO-3) are important intracellular tight junction proteins, linking the cell cytoskeleton to the transmembrane tight junction proteins.

Whereas occludin and junction adhesion molecule have a regulatory role, claudins are transmembrane proteins mainly responsible for the intestinal barrier function. A central regulator of this epithelial barrier is the intestinal microbiota [21]. The importance of intact epithelial TJ is demonstrated again in inflammatory bowel diseases (IBD) [16],[22]. For example, investigations in patients with Crohn's disease (CD) indicated impaired TJ complexity in sigmoid colon biopsies accompanied by a reduced expression of sealing claudin-3, −5 and −8 and occludin as well as a re-distribution of claudin-5 and −8 off the TJ [23]. Analogous changes were observed in ulcerative colitis (UC), comprising down-regulation of claudin-1 and −4 and occludin, but up-regulation of pore-forming claudin-2 [24].

The intestinal epithelium is renewed approximately every 5 days in humans due to proliferation and differentiation of multipotential stem cells located in the crypts of Lieberkühn [25]. At the tips of the villus and epithelial surface in the colon the fully differentiated cells undergo apoptosis and are extruded into the lumen. Intestinal stem cells can differentiate into 4 cell linages, namely enterocytes, enteroendocrine cells, mucus producing goblet cells and Paneth cells which are only found in the human small intestine [26]. Goblet cells secrete mucin which is heavily glycosylated and polymerized into a enormous net-like structure. Mucin 2 is the major component of secreted mucin in the large and small intestine and plays a key role in keeping intestinal microbes at a distance from the epithelial surface.

Colonization by commensal intestinal microbiota is limited to an outer "loose" mucus layer, and interacts with the diverse oligosaccharides of mucin glycoproteins, whereas an "inner" adherent mucus layer is largely devoid of bacteria [27]. In both experimental models of IBD and in humans the mucus layer becomes more permeable to bacteria and is therefore considered critical etiological factors in this disease and possibly other intestinal disorders [28]. In addition to the surface mucins the apical surface of the epithelium is protected by a glycocalyx made up of membrane tethered mucins. These are also glycosylated and are released upon binding by microorganisms as a defense mechanism to prevent colonization [29],[30].

It has recently been recognized that sites of cell shedding represent a third site for the physical intestinal barrier. The intestinal epithelium is one of the most dynamic in the body with epithelial cells arising from stem cells at crypt bases, migrating to the villus tip in the case of the small intestine and the colonic surface from where they are shed. This cellular extrusion process could potentially compromise the integrity of the epithelium. Recent studies have shown that under physiological healthy conditions epithelial extrusion is triggered by stretching of the epithelial cell, which is detected by activation of the stretch-sensitive cation channel Piezo [31]. This triggers a signal transduction pathway involving the sphingosine 1 kinase, the S1P receptor and Rho kinase [32]. This pathway triggers by a redistribution of proteins from the tight junction to surround the shedding cell and to fill the gap left after extrusion is completed thereby maintaining the barrier [33]. This mechanism appears to be very robust such that the barrier rarely fails at sites of cell shedding.

Inflammatory cytokines such as TNFα usually increase the rate of cell shedding. Under these circumstances, the redistribution of the tight junction does not always seal the gap left by shedding cells. This is frequently observed because more than one epithelial cell is shed from one site leaving a gap that is too large to be plugged by the redistribution of tight junction proteins [34].

**Figure 3:**
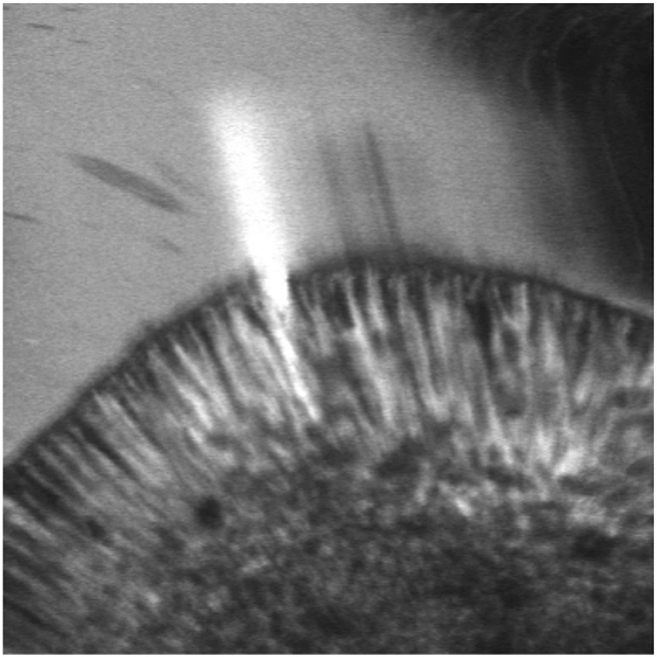
**Cell shedding leading to a temporary epithelial defect** ***.*** Outwardly directed flow of fluorescein through a epithelial defect created by incomplete sealing of a gap created by cell shedding. Image obtained by confocal laser endomicroscopy of a patient with small bowel Crohn’s disease.

An important point is the direction of flow through gaps that are not sealed can be either into or out of the intestinal wall. Local pressure, electrochemical and osmotic gradients determine the direction of flow. In contrast to the tight junction the defects at cell shedding sites are too wide to permit local inwardly directly osmotic gradients to be generated. Instead the direction of flow is determined by the balance of inwardly directed osmotic and electrochemical gradients between the lumen and the subepithelium and outwardly directed hydrostatic gradients generated by hydrostatic pressure and peristalsis (Figure 3). There is a positive hydrostatic pressure in the subepithelium. The direction of flow is thus highly labile and can be readily changed by alterations of osmolarity of the luminal contents as will occur during the mixing as the food travels down the intestine [35].

The mucus components of the gut barrier are fortified by antimicrobial peptides and proteins including lysozyme. Paneth cells produce a range of antimicrobial factors to protect the crypt cells from infection with microorganisms including alpha-defensins, lysozyme and Reg3 proteins [36],[37]. Epithelial cells secrete beta-defensins some of which are upregulated in response to sensing of microbes by pattern-recognition receptors such as Toll-like receptors. Ileal CD is associated with a decreased production of Paneth alpha-defensins in response to sensing of microbes by pattern-recognition of commensal bacteria and pathogens, whereas colonic CD is associated with a reduced expression of HBD1 and a reduced induction of HBD2 and HBD3 also resulting in a reduced mucosal defense [38]. In the mouse, there are more than 20 α-defensins (cryptidins) produced in Paneth cells, but not in neutrophils, and these are processed differently compared to the human system [39]. The Reg3 proteins have recently been shown to play a key role in barrier defense. For example, mouse Reg3g and HIP/PAP in humans has been reported to be bactericidal for Gram-positive bacteria in vitro, and to be important in the spatial compartmentalization of microbes in the intestine [40].

Other structures such as blood vessels, smooth muscle cell layers and components of the enteric nervous system (ENS) contribute to the intestinal barrier by regulating the mucosa and by their capacity to initiate specific defense programs in case of danger [5],[6],[18].

Mammals produce larger amounts of secretory immunoglobulin A (sIgA) in the mucosa reflecting its central role in immunity and protection against pathogens. B cell activation and proliferation leading to antigen-specific sIgA production occurs in the gut associated lymphoid tissue (GALT) via T cell dependent pathways [41]. In addition to the T cell-dependent pathway for IgA production, there is a faster mechanism for generating IgA responses to highly conserved antigenic determinants on commensal bacteria and pathogens [42],[43]. In mice, this involves a specialized subset of B-1 cells that can rapidly produce secretory IgA in the absence of help from CD4+ T cells [44]. This sIgA commonly known as ‘natural’ antibody and appears to be bind several antigens. The repertoire of ‘natural IgA’ is restricted and affinity maturation is limited, although the heavy-chain variable region genes used by IgA-producing plasma cells in the gut are somatically hyper-mutated, to diversify antibody specificity [42]. Equivalent B-1 cells have not been identified in humans but they may have functionally equivalent cells that can colonize the lymphoid tissues and generate natural antibody [45].

Much of the IgA produced in the gut appears not to be reactive to the commensal microbiota. Nevertheless, in germ-free mice sIgA is present at very low levels in the gut and increases substantially soon after colonization with bacteria [46]. sIgA can mediate protection at mucosal surfaces by binding to viruses and bacteria to prevent or inhibit their attachment to and/or invasion of epithelial cells, a process known as immune exclusion [47]. Additionally, sIgA can also interact with antigens presented by intracellular pathogens in endosomes during the pIgR–mediated transport through epithelial cells. Recently we have begun to appreciate its homeostatic roles of sIgA in shaping the intestinal microbiota, preventing mucosal inflammation by immune exclusion, removal of antigen-antibody complexes in the LP and the neutralization of inflammatory mediators.

#### Serotonin, histamine and proteases

Serotonin/5-hydroxytryptamin (5-HT) produced by enterochromaffin cells in the intestine and histamine produced by mucosal mast cells acts as a proinflammatory mediators in the intestine and modulate intestinal permeability [48]-[51]. Our experiments in serotonin reuptake transporter (SERT) knockout mice revealed that absence of SERT aggravates inflammation, because mucosal 5-HT levels increase in an uncontrolled manner. Interestingly, not only experimental colitis is worsened in SERT-knockout mice compared to normal littermates, but also fructose-induced endotoxin translocation and subsequent steatosis in the liver [50].

#### Endogenous cannabinoid system

The plant *Cannabis sativa* has bee used to treat various disorders of the gastrointestinal tract such as vomiting, anorexia, diarrhea, and intestinal inflammation [52]. Recent experimental data in animals indicate now that the intestinal barrier function is regulated *in vivo* through activation of the intestinal cannabinoid type 1 receptor (CB1R). CB1R, but not CB2R, exerts a protective role in colonic permeability as shown in CB1R knockout mice that respond to stress with enhanced permeability compared to normal littermates [53]. Most interestingly, the gut microbiota – and possibly also nutrients – contribute to the regulation of the intestinal barrier via setting the tone of intestinal endocannabinoid system. Cani et al. reported that in obese mice, CB1R is upregulated, and treatment with a CB1R antagonist results in reduced translocation of bacterial antigens such as lipopolysaccharide (LPS) into the systemic circulation [54]. The opposing effects of cannabinoids (either improving or worsening intestinal permeability) might be related to the recent observation that endocannabinoids such as anandamide worsen permeability while phytocannabinoids like cannabidiol and tetrahydrocannabidiol, which might act as (partial) CB1R antagonists, promote protection or recovery from intestinal permeability [52].

### Regulation of intestinal permeability by diet and bacteria

#### Intestinal barrier and the microbiota

The intestinal tract harbors the largest bacterial community associated with the human body, reaching densities of about 10^12^ bacteria per gram of luminal content in the distal colon. Each individual carries up to a few hundred species of intestinal bacteria most of which fall into two dominant phylum, *Bacteroidetes* and *Firmicutes* [55]-[57]. The predominance of certain cornerstone species in the microbiota has been proposed to drive one of three preferred ecological compositions known as enterotypes [58]. Enterotypes appear to be independent of individual host characteristics such as body mass index, age, or gender or geographical location but may be influenced by diet and host genetic backgrounds [59].

The intestinal microbiota is considered to be largely symbiotic in nature and involved in various processes, including the breakdown and absorption of nutrients, the production of vitamins and hormones, and the prevention of colonization by pathogens (Table 2). The gut barrier plays a key role in the avoidance of inflammatory responses to the microbiota and is regulated by a finely tuned network of immune mechanisms for microbial recognition and tolerance to the microbiota [60],[61]. Failure to achieve or maintain this equilibrium between a host and its microbiota has negative consequences for both intestinal and systemic health. Several diseases have been linked to changes in the microbiota populations, or to reduction of the microbiota's diversity, including, atopic diseases, inflammatory bowel disease (IBD), diabetes, obesity, cancer and very recently, even neuropathologies. Some of these pathologies are associated with altered barrier function and increased permeability of the epithelium [6],[7]. The microbiota is implicated in these physiological changes for example via reduced numbers of butyrate producers and butyrate production [62],[63], thereby contributing to the pathophysiology of inflammatory diseases [64]. In IBD, inflammation is also linked to increased abundance of pathobionts such as adherent invasive *E. coli* (AIEC), which can directly damage the barrier to promote inflammatory responses [65],[66].

**Table 2:** **Proposed functions of the human intestinal microbiota**

**I**	Host defense against pathogens and toxins
**II**	Development and maintenance of the intestinal immune system
**III**	Support of digestion by supply of enzymatic capacity

Mucus may serve as binding sites for bacteria enabling them to persist and colonize the surface of the mucus layer. Several species from different phyla can grow on mucus as a carbon source although complete degradation of mucus depends on the concerted action of a consortium of bacteria, due to the high degree of diversity of the mucin oligosaccharide chains and their possible modifications. The normal intestinal microbiota triggers the epithelium for synthesizing mucus sugars, while selected commensal bacteria such as *Akkermansia muciniphila* regulate mucus layers by utilizing mucins as an energy source [67],[68]. When microbiota are not present to degrade mucus e.g. in germ-free rodents, mucus production and degradation are imbalanced, leading to a doubling in the thickness of the mucus layer and swelling of the cecum due to the accumulation of mucus, and the resulting retention of water [69]. Mucin degradation has been associated with bacterial pathogenicity as it erodes the protective mucus layer but in relation to the microbiota it might serve as a host 'prebiotic' to stimulate growth of symbionts and shape the ecology of the microbiota.

#### The intestinal barrier and bacterial pathogens

Many pathogens specifically interact with defined element of the intestinal barrier underlining the importance of bacterial-host interactions in both health and disease. For example, epithelial tight junctions (TJ) can be altered by several pathogens (Table 3). These effects may result from direct modification of TJ proteins such as occludin, or by different kinase mediated effects on the perijunctional actomyosin ring [70]-[85]. Pathogens, as well as usage of antibiotics, might disturb the intestinal mucus layer, either by enhancing mucus degradation, or by inhibiting the normal commensal triggers for mucus production [86].

**Table 3:** **Pathogen interactions with epithelial tight junctions**

*Bacteria*	*Bacterial factors*	*Mechanism of TJ disruption*	*Host targets*	*References*
*H. pylori*	CagA	Cdx2-mediated increase in claudin 2 expression	PAR1	[64]-[66]
	Urease	Phosphorylation of myosin light chain kinase and occludin internalization	MLCK, ROCK	[67]
	Unknown	Rho kinase (ROCK)-dependent loss of TJ claudin-4	IL-1R1, ROCK	[68]
*EPEC*	Map	Cdc42-dependent filopodia and pedestal formation	Cdc42	[69]
	EspM	Activation of RhoA and TJ disruption	RhoA	[70]-[72]
	NleA	Inhibition of host cell protein trafficking through COPII-dependent pathways	COPII	[73]
*V. parahemo- lyticus*	T3SS effectors	Alteration of actomycin ring and TJ disruption	Rho GTPase	[74],[75]
*Salmonella enterica serovar typhimur.*	T3SS effectors SipA, SopB, SopE, SopE2	Filopodia formation and alteration of actomycin ring	Rho GTPase	[76]
*Clostridium difficile*	enterotoxin A and B	Inactivation of Rho family proteins causing degradation of filamentous actin	Rho and Cdc	[77]
*Bacteroides fragilis*	Enterotoxin or fragilysin	Toxin degradation of E- cadherin and alteration of actomycin ring	E-cadherin	[78]
*Vibrio cholera*	HA protease	HA induced cleavage of occludin, alteration of ZO-1 and rearrangement of actin	Occludin	[79]

*Abbreviations*: *TJ* tight junctions, *PAR1* phytochrome rapidly regulated 1 gene, *MLCK* myosin light chain kinase, *ROCK* Rho-associated, coiled-coil containing protein kinase 1, *IL-1R1* interleukin 1 receptor, type I, *Cdc42* cell division cycle 42, *RhoA* ras homolog family member A, *COPII* Rho GTPase, *EPEC* enteropathogenic Escherichia coli. Other explanations see text.

#### Regulation of gut permeability by diet, prebiotics and probiotics

Since we now know about the clinical implications, interest in understanding the regulation of this barrier is growing. Two major regulatory factors could be identified, diet/nutrients/prebiotics, and, secondly, the intestinal microbiota/probiotics. Both are related to life style, which suggests that environmental factors might influence the function of the intestinal barrier and thus gut health [6]. The molecular mechanisms that regulate the epithelial tight junction and the paracellular pathway in response to luminal nutrients as D-glucose are less well defined but have been proposed to involve the cytoskeleton including myosin light chain phosphorylation [87].

The effect of diet on intestinal permeability is dependent on individual factors such as the host’s genetic susceptibility, and also on the intestinal microbiota. For example, the increased gut permeability during metabolic adaptation to high fat diet (HFD) is associated to altered gut microbiota [13]. Dietetic factors that promote increased intestinal permeability and subsequent translocation of bacteria resulting in inflammatory reactions in the liver, the white adipose tissue, the brain, and other organs trigger metabolic diseases such as insulin resistance. This pathophysiological cascade is now accepted to be of major relevance for the development of metabolic diseases including type II diabetes, cardiovascular diseases and non-alcoholic fatty liver disease (NAFLD) or non-alcoholic steatohepatitis (NASH) [88]-[93]. Therefore it is tempting to speculate that tools allowing a safe modulation of the intestinal microbiota such as prebiotic food components or probiotic bacteria might be of great interest for future therapy of intestinal barrier-related diseases.

#### Vitamins

Vitamin A and its derivatives have been shown to regulate the growth and differentiation of intestinal cells, whereas vitamin A deficiency is associated with increased susceptibility to infection in both human and animal models [1]. Vitamin A-deficient diet causes within a few weeks alterations within the commensal bacteria, and impairs the intestinal barrier by changing mucin dynamics and expression of defense molecules such as MUC2 and defensin 6 [94]. Vitamin A deficiency is associated with a decreased small bowel villus height and a reduced disaccharides activity leading to more severe intestinal injury in experimental enteritis [95]. Cross-sectional investigations of children with high rates of subclinical vitamin A deficiency showed that serum retinol concentrations are inversely correlated with intestinal permeability [96]. Apart from vitamin A, also vitamin D seems to play a role for the intestinal barrier. Vitamin D deficiency, a characteristic of IBD, is correlated with the severity of disease [97]. Experiments in vitamin D receptor knockout mice showed that vitamin D deficiency might compromise the mucosal barrier, leading to an increased susceptibility to mucosal damage and an increased risk of IBD [98].

#### Short chain fatty acids (SCFA)

These organic acids comprising acetate, propionate, butyrate and valerate are produced by intestinal microbial fermentation of undigested dietary carbohydrates in the colon. Among them, butyrate plays a particular role for maintaining the intestinal barrier, as shown in IBD, in which deficit in butyrate causes tight junction lesions and finally impaired intestinal permeability [99]. In turn, experiments in a rat model of DSS-induced colitis showed that treatment with butyrate leads to a recovery in transepithelial resistance, which was associated with maintenance of tight junction integrity and inhibition of TNFα release [100].

#### Prebiotics

Apart from the effects of fermentation products of prebiotics such as SCFA, prebiotics by itself might have stabilizing effects on the intestinal barrier. Indeed, prebiotic galactooligosaccharide (GOS) protects against salmonella infections and against barrier impairment in experimental pancreatitis [101],[102]. Most recently, the group of Cani and Delzenne showed that prebiotic fructo-oligosaccharides (FOS) attenuate experimental hepatic steatosis, possibly by modulating the intestinal microbiota or the intestinal barrier function or both [103].

#### Western style diet

A number of animal studies investigated effects of high-fat diets on the composition of gut microbiota and on intestinal permeability [9],[13]. Consistently, energy-rich high-fat diets enhanced intestinal permeability resulting in metabolic endotoxinemia. The Western style diet, which is characterized by a high amount of fat and carbohydrates, induced similar or even more pronounced changes [10]. Moreover, our studies revealed that among dietary sugars fructose plays a particular role with regard to the intestinal barrier. Using TLR-4 mutant mice we showed that the onset of fructose-induced NAFLD is associated with intestinal bacterial overgrowth and increased intestinal permeability, subsequently leading to an endotoxin-dependent activation of hepatic Kupffer cells [104]. Recently, we could also show in a mouse-feeding model that chronic consumption of 30% fructose solution for eight weeks was associated with the loss of the tight junction proteins occludin and ZO-1 in the duodenum and a subsequent increase of bacterial endotoxin in the portal vein [105]. Apart from vitamins and fatty acids, other dietetic factors have been examined such as plant-derived flavonoids, e.g. quercetin present in grapes and onions, which increased epithelial resistance and claudin-4 expression in epithelial cells [106],[107].

#### Probiotics

Several studies report the use of commensal bacteria and probiotics to promote intestinal barrier integrity in vivo [108]-[111] although some studies have been negative or inconclusive. Many studies report enhancement of the intestinal barrier in vitro or protection from barrier disruption by probiotics. For example, the probiotic *E.coli Nissle 1917* (EcN) was shown to prevent barrier disruption caused by infection of T84 and Caco-2 cells with an enteropathogenic E. coli strain [112]. The addition of EcN alone increased expression of ZO-2 protein and redistribution of ZO-2 from the cytosol to cell boundaries in vitro [112]. EcN also increases claudin-14 expression in the epithelial TJ and its effect is mediated via TcpC via PKC-zeta and the MAPK ERK1/2. A similar effect was observed in intestinal epithelial cells isolated from germ-free mice treated with EcN [113]. Metabolites secreted by *Bifidobacterium infantis Y1,* one of the components of the probiotic product VSL#3, leads to an increase in expression of ZO-1 and occludin while reducing expression of claudin-2 leading to enhanced effects on transepithelial resistance and altered ion secretion [114]. Another probiotic strain *Lactobacillus plantarum MB452* (from the VSL3 probiotic) was shown to induce transcription of occludin and cingulin genes [115].

Enteric pathogens often gain access to the body by altering the structure and function of tight junctions to increase permeability of the barrier via the secretion of proteases, which can cleave tight junction proteins or by altering the cytoskeleton [116]. Inflammatory cytokines such as TNFα and IFNγ, which are induced during infection and in IBD, have been shown to increase intestinal permeability in general, although single inflammatory models yielded different results [114] while probiotics and commensals can reverse such inflammatory dysfunctions in human intestinal epithelial cells, e.g. by improving barrier functions or by inhibition of pathogen adherence [115],[117]-[120]. Also synergistic effects between sIgA and probiotics have been described [121].

Importantly, one study has shown that *L. plantarum* can regulate human epithelial TJ proteins in vivo and to confer protective effects against chemically induced disruption of the epithelial barrier in an in vitro model [122]. Administration of *L. plantarum* into the duodenum of healthy human volunteers was shown to significantly increase ZO-1 and occludin in the vicinity of TJ structures [122]. These results suggest that administration of *L. plantarum* can enhance the stability of TJ complexes in humans and may attenuate their disruption by cytokines, toxins and pathogens. Apart from *L. plantarum*, other probiotic *Lactobacillus* strains seem to have protective effects on the intestinal barrier using different in vitro settings or mouse models of disease, namely *L. salivarius strains UCC118* and *CCUG38008, L. rhamnosus GG, Lactobacillus casei strain DN-114 001,* and *L. casei strain Shirota* [123]-[127]. Also the probiotic *E. coli Nissle 1917* up-regulates tight junction proteins such as claudin 14 expression and other components of the intestinal barrier [112],[113],[128].

In summary, imbalances in the composition of bacterial community in the intestine can lead to transient intestinal dysfunctions, barrier modulation, and chronic disease states such as IBD. Understanding how this ecosystem is regulated, e.g. by diet and other exogenous factors, and how to manipulate it is thus essential for disease prevention and therapy.

### Measurement of intestinal permeability

**Figure 4:**
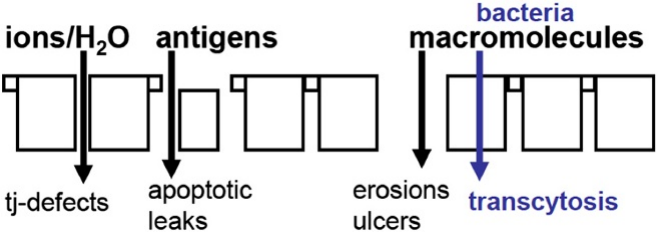
**Intestinal barrier dysfunctions.** Intestinal permeability measurements are determined by the marker molecules used for measurement, since the type of molecules that pass the intestinal barrier depends on the type of lesion.

Intestinal permeability and integrity can be measured in many ways. The techniques used for permeability and integrity assessment vary depending on the setting (in vitro versus in vivo measurements), the species (human or animal models), the marker molecules used for assessment (ions, carbohydrates of different sizes, macromolecules and antigens, bacterial products and bacteria), and the compartments used for measurement of the marker molecules (peripheral blood, portal vein blood, urine). If one focuses just on the epithelial barrier, the flux of molecules is very much dependent on the type of molecules and the type of defects, as illustrated in Figure 4. To measure such dysfunctions, the Ussing chamber is widely used, both for human and animal studies. This *ex vivo* approach to measure intestinal permeability requires intestinal tissue specimens, either biopsies or surgical specimens.

*In vivo* assessment of intestinal barrier function and permeability in humans is currently possible by using intestinal permeability assays, and by the assessment of biomarkers of epithelial integrity such as soluble adhesion molecules, other biomarkers of immunity or inflammation, or bacterial markers like circulating endotoxin (Tables 4 and 5). In addition, histological approaches and scanning electron microscopy analyses have been used in experimental settings. The most relevant methods for assessment of intestinal barrier function and permeability in clinical settings are described in more detail in this chapter.

**Table 4:** **Means for the assessment of intestinal permeability (functional tests, bacteria-related tests)**

*Means*	*Hu*	*An*	*Test molecules*	*Test site*	*Material needed*	*Disadvantages*
***Ex vivo***						
Ussing chamber	x	x	H_2_O, ions, sugars etc.,	site specific	biopsies	invasive
***In vivo – permeability assays***						
Lactulose/mannitol	x	x	oligosaccharides of different MW	small intestine	urine	time consuming
Sucralose	x	(x)	sucralose(comb.)*	colon	urine	time consuming
Sucrose	x	(x)	sucrose(comb.)*	stomach	urine	time consuming
PEG4000/400	x	(x)	polyethylene glycols	whole intestine	urine	time consuming
51Cr-EDTA	x	x	51Cr-EDTA	whole intestine	urine	radio-activity
***In vivo – bacteria-related***						
LAL assay	x	x	endotoxin (LPS)	whole intestine	plasma	assay limitation
EndoCAb	x	x	anti-LPS antibodies	whole intestine	serum	only in acute phase
D-lactate	x	x	bacterial lactate	whole intestine	plasma	low specificity
Butyrate production	x	x	BPB (PCR)	colon	feces	special labs, limited data
Hemolysin test	x	x	pathogens (cell culture)	colon	feces	special labs, limited data
Inner colon mucus	x	x	quantification of bacteria	colon	biopsies	invasive,limited standardization
Liver steatosis	x	x	fat content in the liver	whole intestine	MRT, US	expensive unspecific
Breath tests	x	x	fat content in the liver	whole intestine	GC/MS	unclear specificity

*Abbreviations*: *Hu* suitable for the human system, *An* suitable for animal models, *51Cr-EDTA* chromium labeled EDTA, *BPB* butyrate-producing bacteria, *EndoCAb* circulating endotoxin core antibodies, *GC* gas chromatography, *LAL* limulus amebocyte lysate assay, *LPS* lipopolysaccharide, *MRT* magnetic resonance tomography, *MS* mass spectroscopy, *PEG* polyethylene glycols, *US* ultrasound. *in combination with Lactulose/mannitol test.

**Table 5:** **Means for the assessment of intestinal permeability (biomarkers, histology)**

*Means*	*Hu*	*An*	*Test molecules*	*Test site*	*Material needed*	*Disadvantages*
***In vivo – biomarkers of epithelial cell damage***						
Citrulline	x	x	endogenous ep product	small intestine	plasma	
FABP	x	x	endogenous ep marker	site- specific	plasma	only in acute phase?
αGST	x	x	endogenous ep enzyme	n.a.	plasma, urine	only in acute phase?
Claudin-3	x	x	ep tight junction protein	n.a.	urine	limited data
***In vivo – other biomarkers***						
Fecal calprotectin	x	(x)	neutrophil release product	colon	feces	unspecific marker of gut inflammation
α1-anti- trypsin test	x	(x)	endogenous amino acid	small intestine	feces/ serum	unclear specificity
sIgA	x	x	IgA (ELISA)	whole intestine	serum	low specificity
***In vivo – histological approaches***						
Tight junction expression	x	x	RNA (qPCR), Western blot	site- specific	biopsies	invasive
Goblet cell analysis	x	x	histology	site- specific	biopsies	invasive
Shedding of epithelium	x	x	histology	site- specific	biopsies	invasive
Paneth cell loss**	x	x	histology	site- specific	biopsies	invasive
Defensins			RNA (qPCR), Western blot	site- specific	biopsies	invasive
Mucus analysis***			histology/ staining	site- specific	biopsies	invasive

*Abbreviations*: *αGST* α-glutathione S-transferase, *ep* epithelial, *FABP* fatty acid binding protein, *n.a.* not applicable, *qPCR* qunatitative PCR, see also Abbreviations in Table 4. **Ref. Nr. 226; ***Ref. Nr. 227.

#### The Ussing chamber

**Figure 5:**
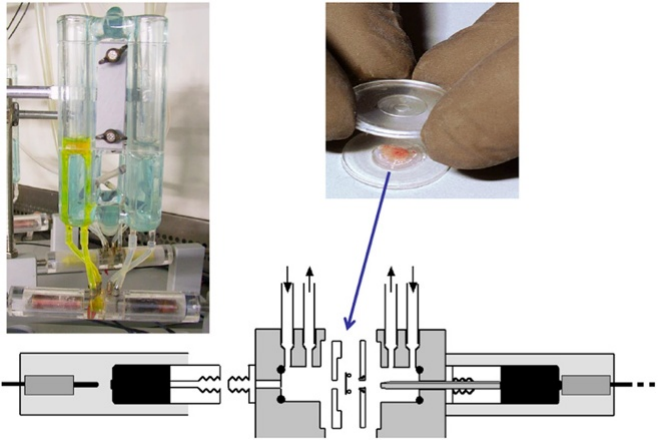
**The Ussing chamber.** Upper left: Ussing chamber equipment. Upper right: Mounting a tissue specimen in a chamber for measurement. Lower panel: schematic view of an Ussing chamber setting. For details see text.

The Ussing chamber allows the measurement of short-circuit current as an indicator of active ion transport taking place across the intestinal epithelium. Basically, the chamber consists of two halves that are mounted together containing the tissue specimen with the apical side isolated from the basolateral side. The two half chambers are filled with equal amounts of Ringer solution (Figure 5). The active ion transport produces a potential difference across the epithelium (V_EP_). The voltage difference generated is measured using two voltage electrodes that are placed as near as possible to the tissue/epithelium. The spontaneous voltage is cancelled out by injecting a counter current using another two current electrodes that are placed far away from the epithelium. This current externally injected is called short-circuit current (Isc) and is the exact measure of net ion transport taking place across the epithelium. The transport of ions through the epithelium, in particular the secretion of chloride, plays an important role in the gut, and is paralleled by water transport [15],[129].

Recent studies showed that luminal factors such as nutrients, bacteria, and bacterial products including probiotics can restore intestinal permeability previously impaired by infections or chronic inflammation, as assessed in the Ussing chamber [16],[130],[131]. In addition, also glutamine is an important luminal component thought to preserve intestinal barrier function, although the molecular mechanism beyond its role as a metabolic fuel is not yet established [132]. Most importantly, malnutrition is associated with increased intestinal permeability, as shown in liver cirrhosis patients, suggesting that nutrients are needed to maintain normal barrier function in the intestine [133]. The Ussing chamber technique is well established since many years and has been successfully used for both human and animal studies. Limitations of Ussing chamber measurements are the invasiveness because of the need for fresh intestinal tissue, and the lack of correlations between Ussing chamber data and other permeability assays raising the question of which aspects of the intestinal barrier can be assessed by this approach.

#### Permeability assays

Permeability assays usually use oligosaccharides of large size, e.g. lactulose or high MW-PEGs of 1500 or 4000 kD, and sugars of small size, e.g. mannitol, L-rhamnose, or low MW-PEG of 400 kD, or other indigestible probes such as 51Cr-EDTA that are administered orally. The large size molecule is thought to cross the paracellular intestinal pathway only if the intestinal barrier function is compromised. In case of barrier function loss such probes cross the intestinal barrier, appear into the circulation and can be detected in urine after renal excretion. The small size molecule is thought to traverse the intestinal barrier freely, independent of barrier function loss, and is affected in the same way as the large molecular probe by the pre- and postmucosal confounders like as gastric dilution, gastrointestinal motility, bacterial degradation, and renal function. Therefore, the ratio of the urinary concentration of both molecules measured after 5–6 h would more accurately reflect the paracellular passage across the intestinal barrier than isolated measurement of urinary oligosaccharides. The “active” test results depend on the test probe size, the way of absorption (passive or active transport in the intestine), the site and velocity of absorption, and the kinetics of distribution into different body compartments. Alternatively to this “active” assessment of the barrier, “passive” assessment of the barrier is possible by the quantification of luminal compounds such as endotoxins and bacterial fermentation in plasma as markers for barrier function integrity. The advantage of this approach is that no time-consuming urine collection is needed. On the other hand, the substances are not always easily measured because of technical limitations (e.g. endotoxin assays) or hepatic metabolism [17],[134].

Laboratory analysis of urine samples is usually performed using high pressure liquid chromatography (HPLC) or liquid chromatography in combination with mass spectrometry (LC/MS). Since some of the saccharides, as lactulose, can cause increased intestinal motility, the administered dose should be kept as low as possible. Permeability assays are usually useful only for assessing small intestinal permeability, since lactulose is degraded by bacteria in the large intestine. To evaluate whole intestinal permeability, non-degradable probes such as sucralose or erythritol, which remain unaffected by bacteria in the colon, are added to classical DST, resulting in the so-called triple sugar test. The lactulose excretion over 24 h (likely to represent only small intestinal permeability), subtracted from 24-h sucralose excretion, is considered to give an isolated measure of colonic permeability [134]. Other studies focused on measurement of gastroduodenal permeability, have used sucrose or glucose as test substances. Sucrose is rapidly degraded by sucrase, an enzyme secreted in large amounts by mature enterocytes in the duodenum. Therefore, enhanced plasma or urinary levels of sucrose are thought to reflect only permeability of the stomach and proximal duodenum [135]. Glucose is even independent of sucrose digestion. Most recently, both approaches have been combined to assess intestinal permeability at different sites. The "multi sugar test" is based on administration of sucrose, lactulose, sucralose, erythritol, and rhamnose simultaneously in order to assess gastro-duodenal, small intestinal and large intestinal permeability in humans [136].

Increased permeability for saccharides has been reported in patients with CD [137],[138], celiac disease [139], adverse reaction to food [140],[141], and in critically ill patients or patients undergoing major surgery [142],[143]. In contrast to lactulose and mannitol, PEGs have the advantage of being inert and can therefore be used to measure both small and large intestinal permeability. They have been used successfully to assess permeability changes in patients with irritable bowel syndrome [144], pancreatitis [145], liver cirrhosis [146], and intestinal ischemia reperfusion injury [147]. Some studies have reported increased colorectal permeability for 51Cr-EDTA in patients with IBD [148]. However, the tests have never gained a place in everyday practice for diagnosis and follow up of such patients groups, mainly because the test is impractical in use and detection methods are complex and not widely available.

Some studies, however, have shown that such permeability assays in intensive care patients have pitfalls. Firstly, decreased motility and altered clearance of the different sugars as a result of renal dysfunction is a complicating factor in these patients. Secondly, the use of mannitol appeared to be unsuitable in patients receiving red blood cell transfusion, since mannitol is used in the storage solution of bank blood [17].

#### Bacteria-related markers

##### LPS measurement

Despite well-known technical limitations of the assay, resulting from the low levels detectable in peripheral blood, several studies have successfully used LPS assays to show endotoxemia, mostly in patients with sepsis [149]. Enhanced levels of LPS were found also in patients with obesity and metabolic syndrome [150],[151], which might indicate bacterial translocation from the gut lumen to the circulation as a consequence of intestinal barrier function failure. While LPS can be quite easily measured in portal vein blood in animals, it remains a challenge to measure LPS in peripheral blood in humans and it requires careful standardization of the measurement.

##### Circulating endotoxin core antibodies (EndoCAb)

Alternatively to the measurement of endotoxin, which yields best results if measured in portal vein plasma, measurement of circulating EndoCAb allowing the quantification of immunoglobulins (IgG, IgM and IgA) against the inner core of endotoxin have been proposed for the acute phase of intestinal barrier damage. This inner core consists of a hydrophobic part, lipid A, which is attached to a core oligosaccharide. Lipid A is highly conserved across the whole range of Gram-negative microbiota. Moreover, it is this part that is considered responsible for endotoxin toxicity. Several studies showed decreased EndoCAb levels postoperatively, accounting for the degree of exposure to endotoxin [152],[153]. Thus, consumption of these circulating immunoglobulins following translocation of gut-derived endotoxins can be used to acquire indirect information on the intestinal epithelial barrier function. The approach is so far limited by the fact that it has been performed successfully only in postoperative patients but not in patients with chronic diseases.

*Plasma D-lactate* level have been originally proposed as a marker for diagnosis of bacterial infections, since D-lactate is a fermentation product produced by many bacteria including those present in the human gastrointestinal tract. Low circulating levels of D-lactate are found in healthy individuals, but in case of intestinal barrier function loss, these levels will rise as a consequence of increased translocation across the intestinal mucosa. Various studies proposed a relationship between plasma D-lactate and intestinal permeability, e.g. in patients undergoing open aortic surgery and ischemic colonic injury. However, results should however be interpreted cautiously where there is bacterial overgrowth since the augmented presence of bacteria could result in increased fermentation of undigested carbohydrates to D-lactate. Therefore, the usefulness of plasma D-lactate as marker for colonic barrier function in man is a subject for future research [17].

##### Fecal butyrate concentrations

Generation of SCFA such as butyrate depends on prebiotic and other dietetic factors as well as on the composition and activity oft he intestinal microbiota. It has been shown that butyrate decreases bacterial translocation in cells models [154] and modifies the expression of the tight junction proteins claudin-1 and claudin-2 in favor of a barrier preservation [99],[155]. Therefore, butyrate deficiency can be taken as an indirect indicator of impaired intestinal barrier function.

Bacteria-derived *hemolysin* is a pro-inflammatory toxin that can impair the intestinal barrier. Conditions leading to enhanced hemolysin concentrations in the intestine will enhance intestinal permeability [156]. However, butyrate and hemolysin assays are poorly established for permeability assessment so far.

##### Assessment of fatty liver disease

Translocation of bacterial or bacterial products such as LPS from the intestine to the liver has been proposed as trigger for liver inflammation and fatty liver disease [151]. We could show that LPS translocation indeed induces hepatic steatosis in mice suggesting that enhanced intestinal permeability is associated with fatty liver disease assessed by histological examination, magnetic resonance tomography or sonography [104]. Alternatively, fatty liver disease can be assessed by a combination of three volatile bacterial compounds exhaled by a breath test and analyzed by gas chromatography–mass spectrometry [157]. It has to be considered that LPS is likely not the only mechanisms that might cause fatty liver disease, limiting the specificity of this approach.

##### Analysis of intestinal mucus for bacterial content

Most recently, it has been shown that under conditions characterized by an impaired intestinal barrier, luminal bacteria enter the inner colon mucus normally impenetrable for the commensals [28]. Therefore, measurement of bacteria in the inner colon mucus of biopsies could serve as a novel marker of intestinal barrier function and permeability. However, a better standardization of this analysis is wanted to establish it as a novel permeability assay.

The bacteria-related markers are clearly less established as markers for intestinal permeability in humans compared to the classical permeability assays; however, the fact that they likely reflect different characteristics of the intestinal barrier, is of great interest and might become of more relevance in future.

#### Biomarkers of epithelial cell integrity

*Plasma levels of citrulline,* an amino acid not incorporated into proteins, but produced by small intestinal enterocytes from glutamine have been proposed as a marker of functional enterocyte mass. Loss of small bowel epithelial cell mass results in impaired intestinal permeability and in declined circulating levels of citrulline, as is shown in haemopoietic stem cell transplant recipients suffering from severe oral and gastrointestinal mucositis following intensive myeloablative therapy [158]. More recently, citrulline was established as a valuable marker for chemotherapy-induced mucosal barrier injury in pediatric patients [159]. Most interestingly, sensitivity and specificity seem to be better for the citrulline assay compared with sugar permeability tests [160].

*Fatty acid binding proteins (FABP)* are small (14–15 kDa) cytosolic water-soluble proteins, present in mature enterocytes of the small and large intestine. Their function is the transport of fatty acids from the apical membrane of the enterocyte to the endoplasmic reticulum where biosynthesis of complex lipids occurs. Three types of FABP are present in the gut; intestinal FABP (I-FABP) found predominantly in the jejunum – less in the colon, liver FABP (L-FABP) found in liver, kidney and intestine, and ileal bile acid binding protein (I-BABP) exclusively present in the ileum. FABP can be measured sensitively in both plasma and urine using an enzyme-linked immunosorbent assay (ELISA). Basal levels of FABP have been reported to reflect the physiological turnover rate of enterocytes, whereas elevated levels indicate intestinal epithelial cell damage. Elevated circulating or urinary FABP levels were reported in patients with intestinal ischemia, systemic inflammatory response syndrome (SIRS) and necrotizing enterocolitis [161]-[163]. Moreover, FABPs have been established as markers of the intestinal barrier function with prognostic relevance in patients with liver transplantation [164], and for disease activity in celiac disease [165],[166]. Since FABP are differentially expressed along the intestinal tract, measurement of specific FABP could be a promising tool to provide information on disease localization [17]. However, data are limited for changes in chronic diseases such as IBD or metabolic disorders.

*Glutathione S-transferases (GSTs)* are involved in cell protection, antioxidation and detoxification of toxic and foreign compounds within the cell by conjugating them to glutathione. The GST family consists of four subgroups displaying tissue variation; αGST, μGST, πGST and θGST. Whilst μGST, πGST and θGST are present in cells of various organs, αGST is predominantly present in liver, kidney and intestine and has been proposed as a potential marker for, amongst others, intestinal epithelial cell damage [17]. Several studies reported that mesenteric ischemia could reliably be predicted by plasma αGST levels in patients suspected for acute mesenteric ischemia [167],[168]. However, increased plasma or urine levels of αGST can indicate intestinal damage as well as liver and kidney damage, because αGST is expressed in epithelial cells of all these organs. Therefore, this test might be useful for assessment of intestinal damage when isolated intestinal damage is suspected.

*Tight junction (TJ)* status can be assessed as a marker for paracellular barrier integrity loss. In particular, claudins are transmembrane epithelial proteins being mainly responsible for intestinal barrier function. The group of Schulzke an co-workers showed a disturbance of the barrier function which was accompanied by a down-regulation of claudin-1, 3, 5, 7 and 8, and an up-regulation of claudin-2, a pore-forming claudin, together with a re-distribution of claudin-5 and −8 off the tight junction domain of the enterocytes in intestinal biopsies of patients with CD [23]. Recent studies showed a strong relationship between intestinal tight junction loss and urinary claudin-3 levels in both a rat hemorrhagic shock model and in patients suffering from IBD or necrotizing enterocolitis, or undergoing major surgery, thereby suggesting that measurement of urinary claudin-3 can be used to some extent as non-invasive marker for intestinal tight junction loss [17].

#### Biomarkers of intestinal inflammation and intestinal immunity

##### Fecal calprotectin

A broad range of pathologies can lead to intestinal inflammation such as neoplasia, IBD, IBS, infections, autoimmune diseases, allergies, intestinal hypoperfusion, and selected drugs like non-steroidal anti-inflammatory drugs. Generally, defects or increased permeability of the mucosal barrier will cause intestinal inflammation in response to the enormous number of bacteria present in the bowel. Recruitment of leukocytes into the intestinal wall is important in the pathogenesis of intestinal inflammation. Activated neutrophils infiltrate the mucosa and their products can be detected in feces. Numerous neutrophil derived proteins present in stool have been studied, including calprotectin, lactoferrin, and elastase. The most promising marker is calprotectin, because of its remarkable resistance to proteolytic degradation and its stability in stool kept at room temperature for at least seven days [169]. It constitutes about 60% of the soluble proteins in human neutrophilic cytosol and is also found in monocytes, macrophages, and ileal tissue eosinophils. It is released during cell activation or cell death and has antiproliferative, antimicrobial, and immunomodulating functions [169],[170]. Fecal calprotectin is nowadays used in clinical practice to evaluate disease activity in the follow-up of patients treated for active IBD and can be easily performed [171].

Apart from calprotectin, other markers of intestinal immunity such as *secretory IgA* [172] and *defensins* have been proposed as markers of intestinal permeability. Whereas secretory IgA has been examined in patients with celiac disease, defensins have been analyzed mostly in patients with IBD [173]. More recently, fecal human β-defensin-2 has been suggested as a marker for intestinal permeability in neonates [174].

Since many years, the macromolecules *ovalbumin*, which is measured in the serum, and *FITC-labeled dextran* that is uptaken in the ileum and transported further to the mesenteric lymph nodes, have been used as markers for small intestinal permeability. Likely, such high-MW markers indicate different qualities of the intestinal barrier than oligosaccharides, but direct comparisons of the different tracers are lacking. On the other hand, their value remains unclear, because in vivo data in humans are scare. Most experiments using ovalbumin or FITC-labeled dextran have been made either in rodents or in Ussing chambers.

##### Histological approaches

**Figure 6:**
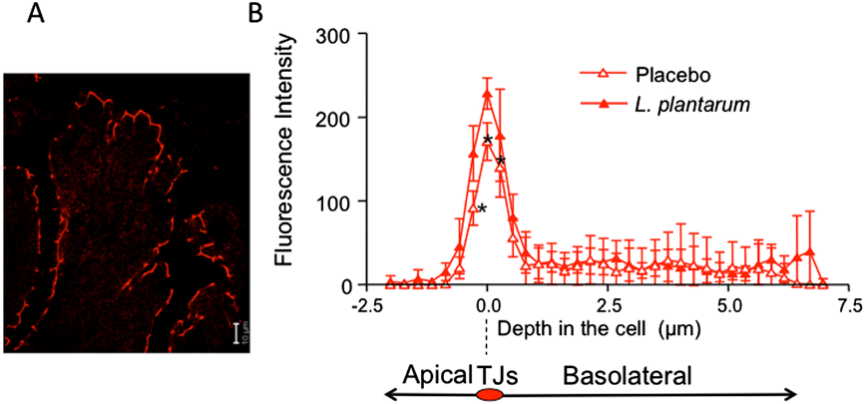
**Tight junctions in the intestine.** This figure is based on previously published data [115] and shows fluorescent staining of occludin in a tissue section perpendicular to the cell surface of the epithelium **(A)**. The fluorescence intensities of 3 different uniform areas per section were plotted as a function of cell location using the peak fluorescence signal from the tight junction region to align each intensity profile **(B)**. Administration of live L. plantarum to humans significantly increased the fluorescent staining of occludin in the tight junction (P < 0.05 for sections indicated *).

Altered tight junction composition can lead to changes in epithelial permeability. Changes in tight junction proteins can be quantified in histological tissue samples by confocal analysis of uniform Z sections perpendicular to the cell surface of the epithelium (Figure 6). Recently this technique was used to measure changes in tight junction staining in human duodenal tissue and in vitro epithelial cell monolayers following administration of a probiotic [122].

### Intestinal permeability – a new target in health and disease?

A number of different diseases comprising intestinal and extraintestinal diseases have been found to be associated with alterations in the intestinal barrier and increased permeability, respectively (Table 6). Among these, IBD and IBS, critical illness, and – more recently – obesity and metabolic diseases have experienced increasing attention and therefore they will be discuss in this chapter in more detail. Other diseases such as celiac disease need to be mentioned as an example of a disease related to intestinal permeability [175],[176]. The realization that the barrier is so important, raises the question of what can disrupt the barrier. Even though no final conclusions can be drawn, it became more and more evident that besides nutrients acting as down-regulators of tight junctions or as histone deacetylase (HDAC) inhibitors, also viral infections, toxins, hypoperfusion of the gut play a role (Table 7). Lifestyle factors such as living place (farming/country site or urban environment), exercise and drug usage seem to play an important role as well, and they offer new approaches for improving gut barrier function [4],[6].

**Table 6:** **Diseases related to intestinal permeability**

*Intestinal*	*Extraintestinal*
Gastric ulcers	Allergies
Infectious diarrhea	Infections (e.g. respiratory)
Irritable bowel syndrome; functional GI diseases	Acute inflammation (sepsis, SIRS, MOF)
Inflammatory bowel disease, Celiac disease	Chronic inflammation (e.g. arthritis)
Cancer (esophagus, colorectal)	Obesity-associated metabolic diseases (NASH, diabetes type I and II, CVD)

**Table 7:** **Possible causes of impairment of the intestinal barrier**

**Nutritional factors**	Tight junction downregulation
	Histone deacetylase (HDAC) inhibitors
	ENS modulators
**Infections & toxins**	Viral intestinal infections
	Environmental toxins
	Toxic food
**“Hygiene hypothesis”**	Sterile environment
	Lack of farming
**“Lifestyle hypothesis”**	Impaired function and diversity
	of the intestinal microbiota
**Endogenous factors**	Hypoperfusion of the intestine
	Chronic inflammation/autoimmunity

#### Role of intestinal permeability and probiotics in IBD

Intestinal barrier dysfunction is a main feature of CD and UC [1],[16],[138]. Already 20 years ago it was found that increased intestinal permeability precedes clinical manifestations of CD, but is insufficient to cause disease suggesting other factors being involved [137],[177]. Leak flux diarrhea and a facilitated uptake of noxious antigens are the two consequences resulting from an impaired epithelial barrier. Barrier perturbations in IBD comprise alterations in epithelial TJ, i.e. a reduced number of horizontal TJ strands and an altered TJ protein expression and subcellular distribution. Recently, prion protein, a ubiquitous cellular glycoprotein being involved in cell adhesion, was found to be dislocated in IBD supporting the concept that disrupted barrier function contributes to this disorder [178]. Moreover, increased incidence of apoptotic events, epithelial cell shedding, as well as erosions and ulcerations can add to that leakiness [179].

These barrier defects are attributed to enhanced activity of pro-inflammatory cytokines like TNFα, INFγ, IL-1β and IL-13, which are highly expressed in the chronically inflamed intestine. They can be detected in vivo in humans by fluorescein leakage analysis and confocal laser endomicroscopy. Crucially, increased cell shedding causing microerosions and barrier loss as assessed by confocal laser endomicroscopy predicts relapse in CD over a 12 month period [35].

Although the etiology of IBD is far from being clear, chronic inflammation is believed to result from an inadequate immune response as a consequence of genetic predisposition as well as changes in, and altered responses to the intestinal microbiota. On the other hand, an insufficient mucosal response to bacterial stimuli results in an insufficient immune response towards intestinal pathogens. The detailed characterization of barrier defects offers the opportunity to consider and test therapeutic interventions. Beside cytokine antagonists, different plant compounds and probiotics have been shown to stabilize the barrier function by affecting TJ protein expression and distribution [16]. Among the plant compounds, Kiwifruit extracts as well as different polyphenols have been found to exert anti-inflammatory effects in models of IBD and in human disease [180]-[182].

The first reports on beneficial effects of probiotics in IBD was on *E.coli Nissle 1917* supporting maintenance of remission in patients with UC [183],[184]. The next important finding was that the probiotic mixture VSL#3 reduces and protects against pouchitis in patients with UC [185],[186]. Since then, more than 80 RCT have been published showing beneficial effects of probiotics in adults and children with UC, but hardly in CD. For example, VSL#3 seems to be effective not only in pouchitis, but also in mild to moderate UC not responding to conventional therapy [187]. More recently, the positive findings could be extended to children with active UC, who improved after treatment with the probiotic *L.reuteri* or VSL#3 [188],[189].

The mechanisms of probiotic effects in IBD is unclear at present, but might involve direct anti-inflammatory effects, e.g. by modulating TLR signaling , or indirect effects such as improvement of the intestinal barrier [190],[191].

#### Role of intestinal permeability and probiotics in IBS

Intestinal barrier dysfunction has been found to play a pathogenic role not only in IBD, but also in IBS [1]. Most importantly, there is evidence now that increased intestinal permeability is related to low-grade inflammation, visceral hypersensitivity and pain in IBS [192]. In diarrhea-predominant IBS (IBS-D), electron microscopy studies showed cytoskeleton condensation and enlarged intercellular spaces between epithelial cells, providing the morphological basis for increased intestinal permeability in IBS. These structural changes were found to correlate both with mast cell activation and symptoms including diarrhea and pain severity [193]. These data confirm and extent earlier observations derived from Ussing chamber experiments showing increased paracellular permeability in colon tissue of IBS patients [194]. The primary cause of the described morpho-functional changes in intestinal permeability remains to be determined. Potential factors include intestinal food allergies, genetic and epigenetic factors, changes in intestinal microbiota. Regardless the cause, mucosal barrier defects determine an increased flow of antigenic substances that challenge the mucosal immune system. Interestingly, several studies have provided evidence of low-grade immune activation and release of inflammatory molecules in IBS which in turn maintain the increase in intestinal permeability [195]. Possibly, the loss of particular TJ proteins such as occludin is a result of increased proteasome-mediated degradation observed in IBS triggered by low-grade inflammation and resulting in increased intestinal permeability [196]. Such data point out the importance of the intestinal barrier in the pathophysiology of IBS and provide evidence for the organic nature of such so-called functional gastrointestinal disorders.

Food, microbiota and bile acids have been discussed as possible inducers of low-grade inflammation and impaired permeability in IBS. In a subgroup of patients, IBS is probably related to food allergy [197],[198]. Apart from external inducers, endogenous triggers such as mast cell-derived histamine, proteases and eicosanoids can increase intestinal permeability, either directly or via stimulation of neurons of the enteric nervous system [194],[199],[200]. Serotonin, another biogenic amine besides histamine, produced by enterochromaffin cells in the gut, is another endogenous trigger of pain, inflammation and increased permeability in IBS [201]. Consequently, LX1031, an oral inhibitor of tryptophan hydroxylase, the key enzyme for mucosal serotonin synthesis has been successful for treatment of patients with non-constipating IBS [202].

In conclusion, there is now substantial evidence that increased intestinal permeability is associated with immune activation and symptoms like pain and diarrhea in IBS. Such knowledge paves the way for the identification of new disease biomarkers and novel therapeutic targets in IBS. Apart from mast cell stabilizers [203] and serotonin antagonists [202], also dietetic approaches [204] and probiotics have been found to be effective to some extent. The value of probiotics for treatment in IBS was debated for long time; however, several recent systematic reviews, guidelines and meta-analyses confirmed, despite all gaps and methodological limitations, that selected probiotics are effective in selected subpopulations of patients with IBS [205]-[209]. In particular, bloating and distension, for with other therapeutic approaches are limited, may improve by probiotic treatment [210],[211]. Most interestingly, some of the probiotic trials demonstrated that the effects are correlated with an improvement of the intestinal permeability [212].

#### Role of intestinal permeability and probiotics in obesity and fatty liver disease

The new concepts on the pathophysiology of obesity and associated metabolic diseases such as NAFLD and NASH, type 2 diabetes mellitus or cardiovascular diseases, stating that such pathologies are related to the intestinal barrier and the intestinal microbiota, derived predominantly from mouse studies. It could be clearly shown that metabolic diseases are linked to increased intestinal permeability and translocation of bacteria or bacterial products like endotoxin from the intestine to the liver and to other tissues [96],[144],[213],[214]. Moreover, it became clear that the microbiota of obese [215],[216] and diabetic [217] individuals differs from that of the healthy, lean population. In the meantime, evidence is growing suggesting that these alterations are of functional relevance.

The altered microbiota in obesity and metabolic diseases contributes to an enhanced harvest of energy from nutrients. In particular, energy harvest from food carbohydrates depends on the microbiota, because specific bacteria found to be increased in obese individuals provide enzymes not expressed by host cells and allowing the digestion of otherwise more or less indigestible carbohydrates [218]-[220]. In type 2 diabetes, the microbiota looses its capacity to generate SCFA from prebiotics [217], which might be a genuine defect or an adaptation to low fiber intake, which has been revealed by several epidemiologic studies [221],[222].

The altered intestinal barrier and the subsequent translocation of small amounts of bacteria or bacterial products is now regarded as one important mechanism causing the low-grade inflammation characteristic for metabolic diseases possibly linked to the subsequent infiltration of organs such as liver, muscle and heart muscle with fat [223]-[226]. Western style diet rich in fat and sugars alters the intestinal barrier in a way resulting in enhanced permeability and elevated endotoxin levels in the portal vein [104],[150],[227].

**Figure 7:**
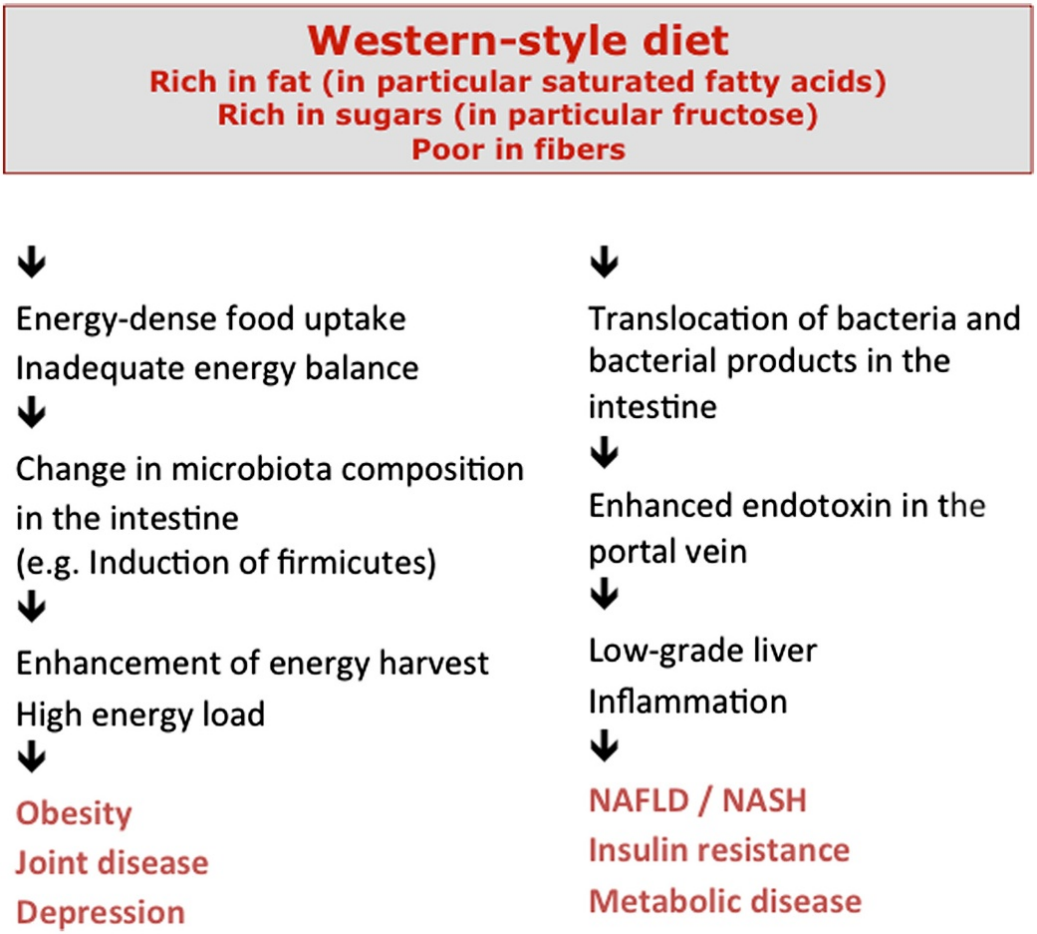
**Current concepts on the pathophysiology of obesity and metabolic diseases related to the gut.** For details see text.

The result of such alterations is enhanced infiltration of tissues with bacteria and bacterial products and subsequent tissue inflammation and fat accumulation, which can be observed first in the liver and later on in other tissues such as muscle or heart muscle [150],[219],[228]. Also in peripheral blood and in adipose tissue bacteria or bacterial products can be observed following feeding with energy-rich Western style diet, an observation that might enable to define new bacterial biomarkers of intestinal barrier dysfunction in metabolic diseases [89],[214]. However, the two alterations, barrier dysfunction and microbiota alteration, are not necessarily linked, but can occur independently [229]. These findings provide a new concept on the pathophysiology of obesity and metabolic diseases that might offer new therapeutic strategies both at the level of diets and of drugs (Figure 7).

Considering these mechanisms it is tempting to speculate that probiotics and prebiotics might have beneficial effects in chronic metabolic disorders. First data derived from experimental studies in mice or from preliminary, human pilot studies indeed point in this direction. For example, possible effects of probiotic bacteria or particular diets on the gut barrier can be studied using organ culture models [230] or feeding models [13],[231]. In humans, overfeeding alters the bacterial composition of the commensal microbiota in healthy individuals in a way that results in increased energy harvest from food [232]. The composition of the commensal microbiota might allow the prediction of weight gain in human individuals at risk like pregnant women [233]. In addition, administration of selected prebiotics or probiotics can improve metabolic alterations in animal models of metabolic liver disease [103],[127] and in obese human individuals [234],[235]. Such data suggest that new therapeutic concepts could be developed in the future to support treatment or prevention of obesity and associated diseases.

#### Role of intestinal permeability and probiotics in the critically ill patient

Not only chronic diseases such as IBD, IBS and metabolic diseases, but also acute intestinal failure and gram-negative sepsis typically observed in the critically ill patient are associated with an impaired intestinal barrier and marked enhancement of intestinal permeability. For that reason, gram-negative sepsis and subsequent MOF is a common cause of death in the intensive care unit (ICU) [236]. Such complications are seen in patients undergoing major abdominal surgery, but also in trauma patients, burn patients and other ICU patients [237]-[239].

Hypo-perfusion of the intestinal tract is regarded as the culprit of such complications. Therefore, such events occur also in patients suffering from acute CVD, acute intestinal ischemia of any cause, and acute enterocyte toxicity, e.g. in the course of chemotherapy [240],[241]. Also under physiological conditions, a hypo-perfusion of the gut can happen resulting in gut dysfunction, e.g. in the course of exercise [242]. This can be assessed by gastric tonometry and by using appropriate biomarkers of enterocyte damage such as intestinal fatty acid binding protein (I-FABP) and ileal bile acid binding protein (I-BABP). In particular, I-FABP, a cytosolic protein in differentiated enterocytes, which can be measured in urine and plasma, has been confirmed as valuable marker for the early diagnosis of intestinal ischemia [243]. Splanchnic hypo-perfusion can be confirmed in healthy men already after cycling for 60 minutes at 70% of maximum workload capacity. A 15 min ischemia causes the appearance of small subepithelial spaces thought to be morphological correlates of an impaired gut barrier [244].

Multiple consequences of enteral ischemia have to be anticipated including mucus barrier loss, bacterial translocation, and enhanced Paneth cell apoptosis causing breakdown of the defensing shield in the intestine [245],[246]. Fortunately, such alterations are rapidly counteracted, e.g. by increased goblet cell secretory activity in the colon [246]. Even the structural defects such as the subepithelial spaces are quickly restored by lamina propria retraction and zipper-like constriction of the epithelium [247]. Such repair mechanisms have been identified in both rodents and man [248].

The classical treatment of loss of barrier functions in the ICU patient is usage of antibiotics directed against gram-negative bacteria and improvement of intestinal perfusion by catecholamines and volume. If pre- or probiotics can support prevention or treatment of sepsis and MOF is unclear at present. Some studies suggested a beneficial effect of selected probiotics and synbiotics on sepsis complications in patients with major abdominal surgery and in immunocompromised patients who underwent liver transplantation; however, the trials were rather small and limited in number [249]-[251]. Other studies performed in patients with severe acute pancreatitis yielded conflicting results. Whereas a few initial studies suggested beneficial effects by treatment with synbiotics [252],[253], one trial reported increased mortality in the verum group [254]. Although this report hat several methodological limitations, the results underline that otherwise harmless probiotics have to be selected and assessed very carefully in severely ill patients similar to pharmaceutical evaluations. Provided that caution is considered, clinical trials are warranted to support the potential use of probiotics in ICU, namely for prevention of antibiotic-associated and *Clostridium difficile*-associated diarrhea, ventilator-associated pneumonia and sepsis [255]. A recent meta-analysis drew the conclusion that the administration of probiotics does not significantly reduce ICU or hospital mortality rates but does reduce the incidence of ICU-acquired pneumonia and ICU length of stay [256].

## Conclusion

Apart from IBD, IBS, metabolic diseases and intestinal failure in critically ill patients, other diseases might be related to the gut microbiota and the intestinal barrier such as celiac disease [175],[176], colon carcinoma [257] or inflammatory joint diseases [258]. Therefore, alteration of the gut barrier seems to have multiple consequences facilitating the onset of a variety of diseases depending on other hits and on genetic or epigenetic constellations, respectively. The growing significance of the gut barrier and bacterial translocation raises the questions of how we can improve gut barrier functions and gut microbiota.

The research on modulation of gut permeability is just starting. On the other hand, a few approaches have been identified among which are dietetic concepts including prebiotics, as well as probiotics, and possibly also fecal transplantation that can be regarded as an unspecific and global probiotic treatment. Indeed, fecal transplantation now enters clinical medicine, after beneficial effects in patients with therapy-refractory Clostridium difficile infection have been reported [259],[260]. Apart from this novel approach, other interventions have been proposed such as particular diets, prebiotics or probiotics (Table 8). Among the diets, some sound promising such as dietary restriction of fat and sugars, or possibly also of poorly absorbed short-chain carbohydrates (FODMAPs) [261]-[263]. Clearly, more intervention trials are urgently needed now to asses the effects of such substances as preventive or therapeutic agents in different populations and diseases, respectively. For these trials, not only known substances (see Table 8), but also new dietetic components and probiotic agents selected according to their beneficial effects on the gut barrier have to be identified and tested.

**Table 8:** **Factors proposed to support the gut barrier**

**Dietetic approach**	Avoidance of high amounts of sugar and fat
	Avoidance of energy-dense Western-style diet
	FODMAP diet
	Prebiotics/fibers
	Glutamine
	Other immune-modulating formula
**Probiotic approach**	Selected probiotics
	Probiotic cocktails (multispecies concept)
	Synbiotics (combination of probiotics and prebiotics)
**Drugs/others**	Short-chain fatty acids (SCFA)
	Metformin
	Quercetin and other flavonoids

To conduct such trials in a scientifically sound way, we need a clear definition and validation of the tools needed to assess gut barrier functions and intestinal permeability. New approaches such as mucus analysis, quantification of translocated bacteria and bacterial products in blood or tissue, and host responses to such alterations, e.g. liver steatosis or fat infection by commensal bacteria, need to be evaluated. Even though European authorities strictly differ between nutrients and drugs, the new tools to modulate intestinal permeability, such as probiotics, prebiotics or other possibly enriched dietetic components, need clear scientific evaluation independent of their legal classification, which can be questioned from a scientific point of view [264],[265]. The fact that such substances can be used both for prevention of disease in the general population and for prophylaxis or treatment of disease in defined subpopulations and thus touch both legal categories of substances, a third category should be considered placed between the two existing ones. This new category could be named for example “functional food” usable for both healthy nutrition and medical intervention and requiring trials based on elements established in part in the health claim regulations and in part in the drug laws. A political effort to establish such a new “functional food” category in the European legislation, similar to the one existing in Japan, would support more scientific efforts as well as substantial business efforts to develop this new exiting area of health research further.

Despite many open questions, intestinal permeability becomes an area of growing interest both in basic science and for clinicians, because it might by a valuable new target for disease prevention and therapy. The expert panel agreed on several conclusions:

- Definition of intestinal permeability and intestinal barrier (see Table 1).
- Assessment of intestinal permeability and intestinal barrier (see Tables 4 and 5).
- Given the importance of the physiological and thus the clinical role of the intestinal permeability there is a need for biomarkers that not only reflect lost functionality of the intestine, but also permeability per se and permeability-related functions such as mucus quality. More research is needed to develop reliable non-invasive, rapid diagnostic means.
- The role of microbiota in the regulation of intestinal permeability also requires additional research.
- Food intake is of importance for the intestinal microbiota composition as well as for intestinal permeability, but to which extent? Apart from dietetic approaches, what else can attenuate negative effects of nutrients? What is the preventive capacity of pre- and probiotics?
- The question of how to define a healthy microbiota needs to be addressed. Can an "unhealthy microbiota" affect intestinal permeability in a negative way?
- Are changes in intestinal microbiota in diseases such as IBS or IBD cause or effect of the disease? Can an "unhealthy" intestinal microbiota impair the mucosal immune system through an excessively permeable mucosal barrier, and thus perturb bowel physiology and sensory perception?
- Can we look at the intestinal microbiota as a novel therapeutic tool to improve intestinal permeability and gut health?

## Authors’ contributions

All authors participated in the round table discussion on which the article is based. The manuscript was written by SCB and revised and extended by all co-authors. All authors read and approved the final paper.

## Authors’ original submitted files for images

Below are the links to the authors’ original submitted files for images. Authors’ original file for figure 1 Authors’ original file for figure 2 Authors’ original file for figure 3 Authors’ original file for figure 4 Authors’ original file for figure 5 Authors’ original file for figure 6 Authors’ original file for figure 7
