# Supplementary figures and images for: Intestinal permeability – a new target for disease prevention and therapy
Source: BMC Gastroenterol. 2014 Nov 18;14:189. doi: 10.1186/s12876-014-0189-7 (PMC4253991; doi:10.1186/s12876-014-0189-7)

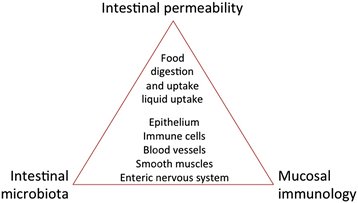

Supplement: Supplementary file 1 — Authors’ original file for figure 1 [file 12876_2014_189_MOESM1_ESM.gif]

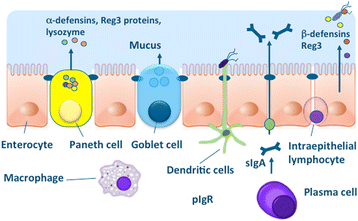

Supplement: Supplementary file 2 — Authors’ original file for figure 2 [file 12876_2014_189_MOESM2_ESM.gif]

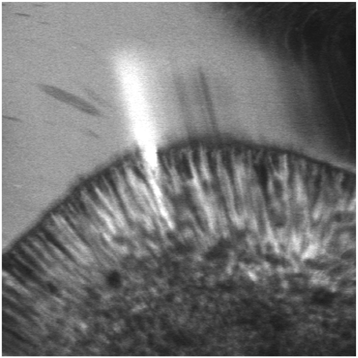

Supplement: Supplementary file 3 — Authors’ original file for figure 3 [file 12876_2014_189_MOESM3_ESM.gif]

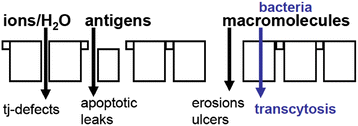

Supplement: Supplementary file 4 — Authors’ original file for figure 4 [file 12876_2014_189_MOESM4_ESM.gif]

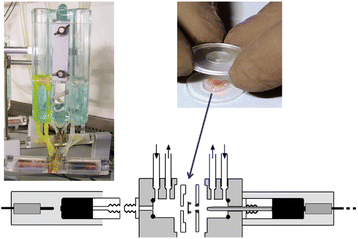

Supplement: Supplementary file 5 — Authors’ original file for figure 5 [file 12876_2014_189_MOESM5_ESM.gif]

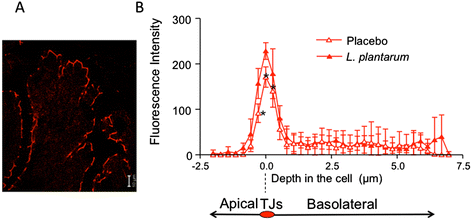

Supplement: Supplementary file 6 — Authors’ original file for figure 6 [file 12876_2014_189_MOESM6_ESM.gif]

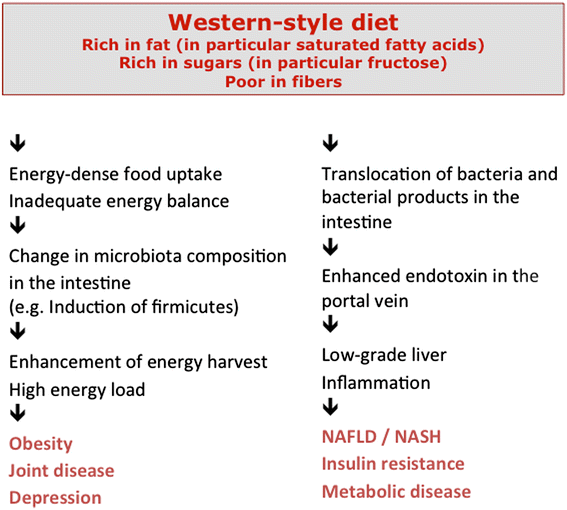

Supplement: Supplementary file 7 — Authors’ original file for figure 7 [file 12876_2014_189_MOESM7_ESM.gif]
